# Polyol Structure and Ionic Moieties Influence the Hydrolytic Stability and Enzymatic Hydrolysis of Bio-Based 2,5-Furandicarboxylic Acid (FDCA) Copolyesters

**DOI:** 10.3390/polym9090403

**Published:** 2017-08-30

**Authors:** Karolina Haernvall, Sabine Zitzenbacher, Motonori Yamamoto, Michael Bernhard Schick, Doris Ribitsch, Georg M. Guebitz

**Affiliations:** 1 ACIB: Austrian Centre of Industrial Biotechnology GmbH, Konrad Lorenz Strasse 20, 3430 Tulln an der Donau, Austria; karolina.haernvall@gmail.com (K.H.); sabine.zitzenbacher@richard-bittner.com (S.Z.); guebitz@boku.ac.at (G.M.G.); 2 BASF SE, Carl-Bosch-Straße 38, 67056 Ludwigshafen am Rhein, Germany; motonori.yamamoto@basf.com (M.Y.); michael-bernhard.schick@basf.com (M.B.S.); 3 BOKU, University of Natural Resources and Life Sciences, Institute for Environmental Biotechnology, Konrad Lorenz Strasse 20, 3430 Tulln an der Donau, Austria

**Keywords:** cutinase, *Thermobifida cellulosilytica*, bio-based, sulfonated isophthalic acid, poly(2,5-furan dicarboxylate)

## Abstract

A series of copolyesters based on furanic acid and sulfonated isophthalic acid with various polyols were synthetized and their susceptibility to enzymatic hydrolysis by cutinase 1 from *Thermobifida cellulosilytica* (Thc_Cut1) investigated. All copolyesters consisted of 30 mol % 5-sulfoisophthalate units (NaSIP) and 70 mol % 2,5-furandicarboxylic acid (FDCA), while the polyol component was varied, including 1,2-ethanediol, 1,4-butanediol, 1,8-octanediol, diethylene glycol, triethylene glycol, or tetraethylene glycol. The composition of the copolyesters was confirmed by ^1^H-NMR and the number average molecular weight (*M*_n_) was determined by GPC to range from 2630 to 8030 g/mol. A DSC analysis revealed glass-transition temperatures (*T*_g_) from 84 to 6 °C, which were decreasing with increasing diol chain length. The crystallinity was below 1% for all polyesters. The hydrolytic stability increased with the chain length of the alkyl diol unit, while it was generally higher for the ether diol units. Thc_Cut1 was able to hydrolyze all of the copolyesters containing alkyl diols ranging from two to eight carbon chain lengths, while the highest activities were detected for the shorter chain lengths with an amount of 13.6 ± 0.7 mM FDCA released after 72 h of incubation at 50 °C. Faster hydrolysis was observed when replacing an alkyl diol by ether diols, as indicated, e.g., by a fivefold higher release of FDCA for triethylene glycol when compared to 1,8-octanediol. A positive influence of introducing ionic phthalic acid was observed while the enzyme preferentially cleaved ester bonds associated to the non-charged building blocks.

## 1. Introduction

There is an increasing interest in replacing petroleum-based raw materials by renewable bio-based resources in polymer production [1,2,3,4]. One of the promising renewable building blocks is 2,5-furandicarboxylic acid (FDCA), an aromatic diacid derived from cellulose or hemi-cellulose [5,6]. The production of polyesters based on 2,5-furandicarboxylic acid [7,8], and polyurethanes based on 5,5′-bihydroxymethyl furil and 5,5′-bihydroxymethyl hydrofuroin [9], has recently been reported. The successful production of an FDCA-based polyester on a large scale, namely poly(ethylene furanoate) (PEF), was reported by Avantium in 2015 [10]. Moreover, recently, the potential of enzymatic synthesis for FDCA-based polyesters has been demonstrated [11,12,13]. PEF is regarded as a new polyester with superior properties [4,14,15]. Nevertheless, polyester properties need to be tuned to match the requirements for different applications. One effective way to tune the chemical as well as physical properties of polymers is to introduce ionic moieties into the polymer. The introduction of ionic moieties can, for example, be used to improve the dyeability of textile fibers, create delivery systems for charged proteins and drugs, shape memory polymers and self-healing polymers, improve the properties of soil-releasing agents and textile-sizing agents, or to increase the hydrolytic degradation rate of polymers when desired [16]. For this purpose, frequently sulfonated monomers are introduced into polyesters, which have been extensively investigated regarding their impact on viscosity, crystallinity, mechanical properties, and hydrolytic stability [17,18,19]. Another promising approach to tune the properties of polyesters is by surface functionalization. The functionalization of polyesters can be done by conventional methods, such as wet chemistry, photo grafting, or plasma treatment, often resulting in the damage of polymer properties, such as mechanical strength. The conventional methods also require high pressure, high temperature, and/or considerable amounts of toxic and hazardous chemicals [20,21]. An alternative approach is functionalization by limited enzymatic hydrolysis, which would be in line with the attempts to render polymer production environmentally friendlier. Enzymatic functionalization is performed under mild conditions and utilizes a renewable and biodegradable biocatalyst. Enzymes create active groups on the surface in a highly specific manner and leave the bulk untouched, thereby leaving the physical properties of the polymers unaffected. Enzymes are therefore suitable biocatalysts for environmentally friendlier functionalization or recycling processes [22,23]. Enzymatic surface hydrolysis has previously been reported for polymers such as polyester, polyamide, and the polyurethanes [24,25,26,27], while the enzymatic hydrolysis of sulfonated PET films was shown to improve water wetting and absorbent properties [28]. We have recently demonstrated the enzymatic hydrolysis of PEF by cutinase 1 from *Thermobifida cellulosilytica* [29], as well as investigated how the polyol structure influences the enzymatic hydrolysis of bio-based 2,5-furandicarboxylic acid (FDCA) polyesters [30], while others have reported on the hydrolysis of the poly(butylene adipate-*co*-butylene furandicarboxylate (PBAF) copolyester by lipase from porcine pancreas [31]. However, the enzymatic hydrolysis of sulfonated furanic polyesters has not yet been investigated, and the impact of the chain lengths of the diol units has not been elucidated. Hence, the aims of this study were to synthesize copolyesters based on 5-sulfoisophthalic acid and 2,5-furandicarboxylic acid with altering alkyl and ether diols to investigate their influence on hydrolytic stability and enzymatic hydrolysis.

## 2. Materials and Methods

### 2.1. Chemicals, Reagents, and Enzyme

Cutinase 1 from *Thermobifida cellulosilytica* was expressed and purified as previously described by Herrero Acero et al. (2011). The colorimetric assay kit for protein quantification was purchased from Bio-Rad Laboratories GmbH (Bio-Rad Laboratories GmbH, München, Germany). Buffer components, bovine serum albumin (BSA), *para*-nitrophenol (p-NP), *para*-nitrophenyl esters (p-NP esters), 2,5-furandicarboxylic acid (FDCA), methanol (HPLC grade), hexafluoroisopropanol (HPLC grade), and potassium trifluoroacetate were purchased from Sigma-Aldrich (St. Louis, MO, USA). Dimethyl sulfoxide (DMSO) was purchased from Merck Millipore (Billerica, MA, USA). All other chemicals and solvents used were purchased from Sigma-Aldrich (St. Louis, MO, USA) at reagent grade and used without further purification.

### 2.2. Synthesis and Characterization of Furanic-Sulfonated Isophthalic Copolyesters

The preparation of Poly(ethylene furanoate-*co*-ethylene sodium sulfoisophthalate) (PEFSI) is described as an example of polyester synthesis. The reagents 5-sodiumsulfoisophthalic acid dimethyl ester, 1,2-ethandiol, and tetrabutyl titanate were mixed and heated up to 180–200 °C for 50 min. The catalyst tetrabutyl titanate was used to a concentration of 100 ppm by mass (as *T*_i_ concentration) relative to the polymer. Methanol was distilled off during the reaction. Afterwards, 2,5-furandicarboxylic acid dimethyl was added to the mixture and stirred at 180–200 °C for 45 min. Again, methanol was distilled off before the temperature of the mixture was increased to 240 °C. In parallel, a vacuum was gradually applied to approximately 1 mbar. This vacuum phase took 30 min. The increase in the viscosity during the reaction was monitored by the continuous measurement of the torque (HEIDOLPH RZR 2052 stirrer, Heidolph Instruments GmbH & Co.KG, Schwabach, Germany) of the stirrer.

For synthesizing the other copolyesters, the same procedure was applied while the diol was altered, resulting in following polymers: poly(butylene furanoate-*co*-butylene sodium sulfoisophthalate) (PBFSI), poly(octanylene furanoate-*co*-octanylene sodium sulfoisophthalate) (POFSI), poly(diethylene furanoate-*co*-diethylene sodium sulfoisophthalate) (PDEFSI), poly(triethylenefuranoate-*co*-triethylene sodium sulfoisophthalate) (PTEFSI), and poly(tetraethylenefuroanate-*co*-tetraethylene sodium sulfoisophthalate) (PTeEFSI).

For analysis by proton nuclear magnetic resonance (^1^H-NMR), the samples were dissolved in dimethyl sulfoxide (DMSO). The 400-MHz ^1^H-NMR spectra of the polyesters were recorded on a Bruker AV 400 (Bruker Corporation, Billerica, MA, USA) spectrometer at 25 °C for 2 min and 45 s. ^1^H-NMR spectroscopy was used to determine the copolyesters’ composition. To determine the dyad sequence distribution, the relative peak intensities of the 2,5-furandicarboxylic acid (FDCA) and 5-sulfoisophthalic acid (NaSIP) dyads were compared, and their peak areas were considered to be equivalent to the dyad quantities. The polyester composition was calculated as follows:

(1) $$\%\mathrm{NaSIP}=\frac{A_{\mathrm{NaSIP}}/H_{\mathrm{NaSIP}}}{A_{\mathrm{NaSIP}}/H_{\mathrm{NaSIP}}+A_{\mathrm{FDCA}}/H_{\mathrm{FDCA}}}*100$$

and

(2) $$\%\mathrm{FDCA}=\frac{A_{\mathrm{FDCA}}/H_{\mathrm{FDCA}}}{A_{\mathrm{NaSIP}}/H_{\mathrm{NaSIP}}+A_{\mathrm{FDCA}}/H_{\mathrm{FDCA}}}*100$$

where *A*_NaSIP_ is the sum of NaSIP proton integrals, *A*_FDCA_ is the sum of FDCA proton integrals, *H*_NaSIP_ is the sum of hydrogens in NaSIP, and *H*_FDCA_ is the sum of hydrogens in FDCA.

The infrared spectra data of the FDCA-based polyesters were obtained using an ATR-FTIR (Attenuated Total Reflectance Fourier Transform Infrared) spectrophotometer (Bruker Tensor 37 FTIR, Bruker Corporation, Billerica, MA, USA). The spectra were recorded in the range of 4000–600 cm^−1^, with air as background signal.

Gel permeation chromatography (GPC) was performed with a conventional GPC apparatus from the Agilent 1100 series (Agilent Technologies, Santa Clara, CA, USA) equipped with columns PSS GRAM (8 × 50 mm), PSS GRAM 30A (8 × 300 mm), PSS GRAM 1000A (8 × 300 mm), and PSS GRAM 1000A (8 × 300 mm) (Polymer Standards Service GmbH, Mainz, Germany) and a refractive index (RI) detector (Agilent Technologies, Santa Clara, CA, USA). For analysis, 100 μL of a 4 mg/mL sample solution were injected. The products were eluted at 85 °C using dimethylacetamide supplemented with 0.5% lithium bromide at a flow rate of 1 mL/min. The GPC calibration was carried out with poly(methyl methacrylate) (PMMA) standard (800–1,820,000 g/mol) from PSS-Polymer Standards Service GmbH (Mainz, Germany).

The glass-transition temperature (*T*_g_) of the polyesters was determined by differential scanning calorimetry (DSC) (Malvern MicroCal, Malvern Instruments Ltd, Worcestershire, UK) according to the standard DIN EN ISO 11357. DSC was carried out over a temperature range of 80 to 200 °C and at a heating and cooling rate of 20 K/min.

The series of bio-based 2,5-furandicarboxylic acid (FDCA)-based polyesters was synthetized with various polyols via a direct esterification and analyzed as described previously [30].

### 2.3. Protein Quantification and Enzyme Activity

The Bradford-based Bio-99 Rad Protein Assay (Bio-Rad Laboratories GmbH, Munich, Germany) with bovine serum albumin as standard was used to determine the protein concentration of the purified enzymes. The protein assay was performed according to the manufacturer’s instruction [32]. The activity of the enzymes was measured by using a photometric assay based on soluble *p*-nitrophenyl esters as previously described by Pellis et al. (2016).

### 2.4. Hydrolytic Stability and Enzymatic Hydrolysis

The copolyesters were solubilized (60 °C, 14,000 rpm, 30 min) in 100 mM potassium phosphate buffer of pH 7.0 to a final concentration of 10 mg/mL and incubated in the presence or absence of 1 µM Thc_Cut1 in an orbital shaker (50 °C, 100 rpm). Samples were taken after 24, 48, and 72 h for the quantification of released 2,5-furandicarboxylic acid and 5-sodiumsulfoisophthalic acid into solution. The polyesters and enzymes were incubated in pure buffer as blank, and all experiments were run in triplicate. Larger molecules and enzymes were precipitated by the addition of ice-cold methanol (1:1 *vol*/*vol*). The samples were centrifuged (Hermle Z300K, Hermle Labortechnik GmbH, Wehingen, Germany) for 15 min at 0 °C and 14,000 g before further HPLC analysis of the supernatant was performed.

### 2.5. Determination of Released Acids

The prepared hydrolysis samples were analyzed by HPLC-UV on a system consisting of a Dionex UltiMate 181 3000 Pump, a Dionex ASI-100 automated sample injector, a Dionex UltiMate 3000 column compartment, and a Dionex UVD 340 U photodiode array detector (all instruments are from Dionex Cooperation, Sunnyvale, CA, USA). The released acids were separated on a reversed phase column XTerra^®^ RP18, 3.5 μm, 3.0 × 150 mm column (Waters Corporation, Milford, MA, USA) using an isocratic method. The method consisted of 8% methanol, 10% 0.1% formic acid, and 82% water, and the flow rate was 0.4 mL/min. The injection volume was 1 µL and the column compartment was set to 40 °C. The expected release products, 2,5-furandicarboxylic acid and 5-sodiumsulfoisophthalic acid, were detected using a UV detector at the wavelength of 254 nm, and were qualified and quantified using external standard calibration curves.

## 3. Results and Discussion

The aim of this study was to investigate the effect of polyols’ structure and sulfonate isophthalic moieties on the hydrolytic stability and enzymatic hydrolysis of various 2,5-furandicarboxylic acid-based copolyesters. A variety of copolyesters was therefore synthetized based on 2,5-furandicarboxylic acid and 5-sulfoisophthalic acid units with various alkyl and ether diols. Hydrolytic degradation under neutral conditions and enzymatic hydrolysis with cutinase 1 from *Thermobifida cellulosilytica* were investigated.

### 3.1. Furanic-Sulfonated Isophthalic Copolyesters

**Figure 1:**
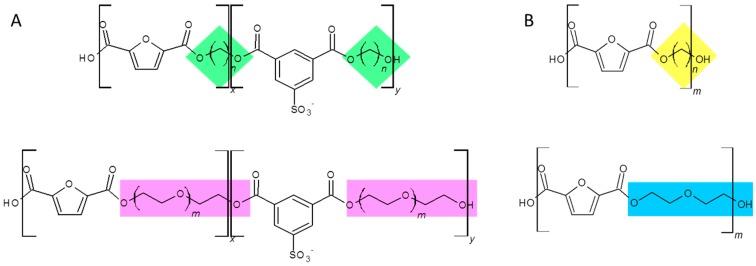
(**A**) Chemical structure of copolyesters based on 5-sulfoisophthalic acid and 2,5-furandicarboxylic acid with altering alkyl and ether diols, with altering alkyl diol (green diamond) and ether diol (pink rectangle), where *n* is 2, 4, or 8, and *m* is 2, 3, or 4. (**B**) Chemical structure of polyesters based on 2,5-furandicarboxylic acid with altering alkyl diol (yellow diamond) and ether diol (blue rectangle), where *n* is 3, 5, 6, 8, 9 or 12.

**Figure 2:**
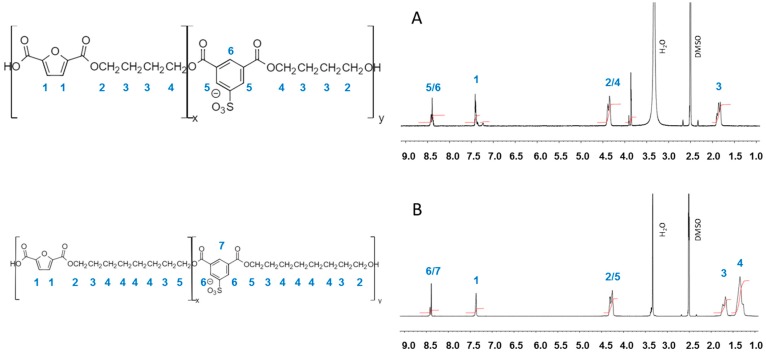

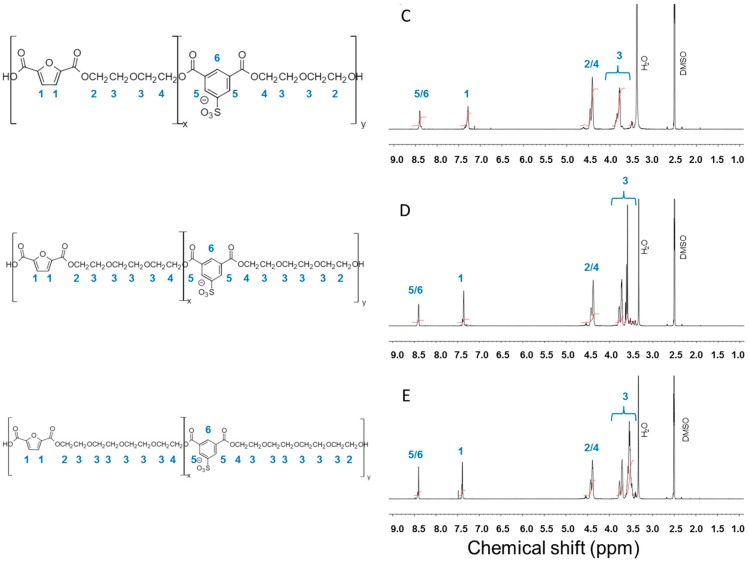
^1^H-NMR spectrum of (**A**) PBFSI, (**B**) POFSI, (**C**) PDEFSI, (**D**) PTEFSI and (**E**) PTeEFSI.

Copolyesters based on 5-sulfoisophthalic acid and 2,5-furandicarboxylic acid with different alkyl and ether diols (Figure 1) were successfully synthesized and their structure confirmed by ^1^H-NMR (Figure 2). The relative areas of the aromatic proton signals appearing at 8.4 ppm and 7.4 ppm for the 5-sulfoisophthalic acid and 2,5-furandicarboxylic acid units, respectively, were measured to estimate the content of the two aromatic monomers in the copolyesters. The copolyester compositions were found to be essentially the same as the composition used in the reaction feeds (Table 1). The complex signals observed in the 3.4–4.4 ppm region arise from ether diol units contained in the diol counterpart of the copolyester. The results are consistent with already published data on 2,5-furandicarboxylic acid-based polyesters [2,3,33].

The molecular characteristics of the copolyesters were estimated by gel permeation chromatography (GPC) (Table 1). The number average molecular weights (*M*_n_) of the copolyesters based on alkyl diols were in the range from 2630 to 5790 g/mol with a polydispersity index (PDI) from 1.3 to 2.3. The *M*_n_ of the copolyesters based on ether diols were slightly higher, ranging from 6830 to 8030 g/mol with a PDI from 1.9 to 2.1. The aim was to produce copolyesters with comparable molecular weights to be able to compare the enzymatic hydrolysis of the different polyesters. The slightly lower molecular weight of the polyester-containing butanediol could be due to unwanted side reactions, as discussed for poly(butylene succinate) (PBS) [34]. A possible solution to overcome this could have been to synthesize the polyester with a modified procedure for the esterification method to get a higher molecular weight [2].

In addition, the copolyesters were synthesized with an excess of diols to ensure that the hydroxyl end groups enhanced the hydrolytic stability, since carboxylic end groups are known to catalyze hydrolysis [17]. A random sequence distribution of the copolyesters is expected due to the two-step polyester synthesis performed. A block structure of the polymer would also result in two different glass-transition temperatures, but only one was observed from each polymer in this study (Table 1).

All copolyesters were soluble in 100 mM potassium phosphate buffer of pH 7.0 to a final concentration of 10 mg/mL except POFSI, which displayed poor solubility.

The microstructure of the copolyesters in potassium phosphate buffer was not determined, but the copolyesters are expected to form nanoparticles in potassium phosphate buffer due to their ionic characteristics. The polyesters with more hydrophobic diols (longer alkyl diols, shorter ether diols) are expected to form bigger particle sizes when compared to more hydrophilic diols (shorter alkyl diols, longer ether diols), resulting in a smaller surface area.

**Figure 3:**
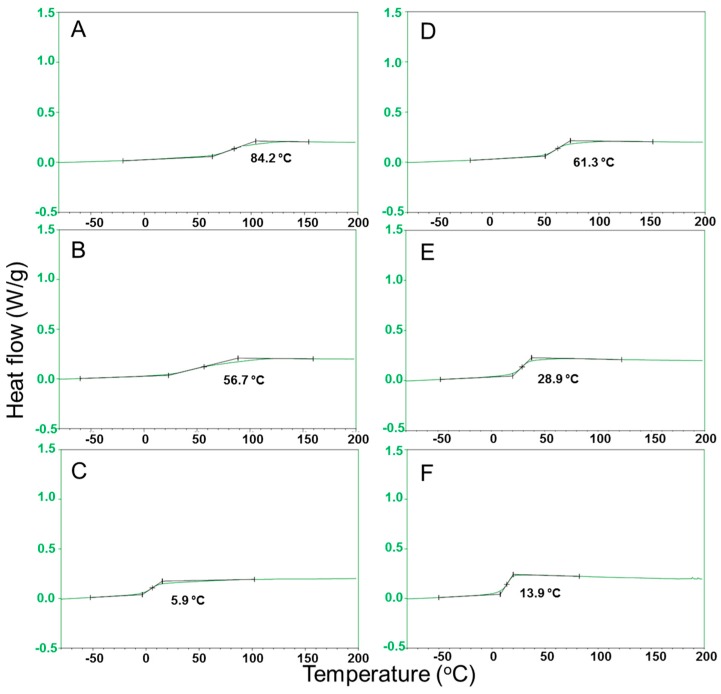
DSC thermographs for the glass-transition temperatures of the melted polymer with a cooling rate of 20 K/min for (**A**) PEFSI, (**B**) PBFSI, (**C**) POFSI, (**D**) PDEFSI, (**E**) PTEFSI and (**F**) PTeEFSI in dimethyl sulfoxide.

The glass-transition temperatures (*T*_g_) of the copolyesters were determined by DSC analyses. The *T*_g_ (glass-transition temperature of a sample cooled from the melt) of the polyesters was in the range from 84 to 6 °C (Table 1 and Figure 3), and no signs of crystallinity were detected. As a result of introducing longer diols into the polyester chain, the glass-transition temperature decreased continuously and did not follow the odd-even effect as also previously reported [8], indicating that the flexibility of the molecular chains was increased with an increasing length of the methylated unit. Previous studies have addressed that, in some cases, the fast crystallization rates of the polyesters did not allow for an accurate measurement of the glass-transition temperature of amorphous polyesters [33,35]. The glass-transition temperatures were in the range previously reported for copolyesters based on a content of 20 mol % of sodium sulfoisophthalate units and (5,5′-isopropylidene-bis(ethyl 2-furoate)) with different alkyl diols [36].

**Figure 4:**
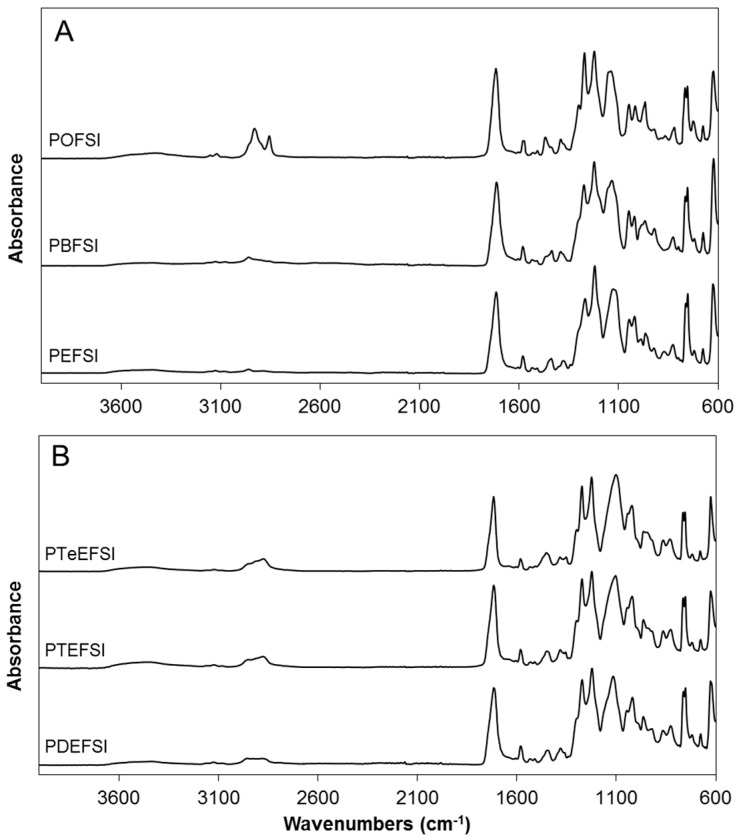
FTIR spectrum of copolyesters based on 5-sulfoisophthalic acid and 2,5-furandicarboxylic acid with altering alkyl (**A**) and ether (**B**) with altering polyols, where the codes represent the different alkyl and ether diols in the copolyesters and where PEFSI is 1,2-ethanediol, PBFSI is 1,4-butanediol, POFSI is 1,8-octanediol, PDEFSI is diethylene glycol, PTEFSI is triethylene glycol, and PTeEFSI is tetraethylene glycol.

The FTIR spectra collected between 4000 and 600 cm^−1^ with the corresponding peak assignments for all polyesters (Table 2 and Figure 4) are in excellent agreement with spectra previously reported for both PET [37] and FDCA-based polyesters with altering polyols [38,39,40] and sulfonated poly(hexamethylene terephthalate) copolyesters [19]. The characteristic absorbance bands of the furan rings in the FDCA-based polyesters, such as the C=C peak, appeared in the range of 1582–1578 cm^−1^, the furan ring breathing around 1020 cm^−1^, and the bending motions associated with the furan ring around 970, 820 and 760 cm^−1^. Also, the characteristic absorbance bands of the C=O of the ester carboxylic group appeared in the range of 1718–1710 cm^−1^, and the C–O peak of the ester carboxylic group around 1270 cm^−1^, depending on the nature of the group directly attached to it. The SO_2_ asymmetric and symmetric stretching vibrations appeared at 1050 and 1130 cm^−1^, and a peak appeared at 753 cm^−1^ arising from the S–O bond. The characteristic bands of the C–H peak appeared around 2900 cm^−1^, and increased with increased hydrocarbon chain length in the polyester as expected. No significant absorption in the OH-stretching region was detected, suggesting that the synthesis resulted in polyesters with high molecular weights, as confirmed by the GPC data (Table 1).

### 3.2. Hydrolytic Stability

**Figure 5:**
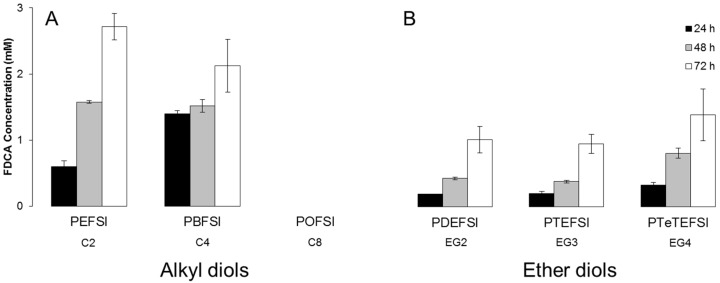
Hydrolytic degradation of copolyesters based on 5-sulfoisophthalic acid and 2,5-furandicarboxylic acid with (**A**) altering alkyl and (**B**) ether diols in 100 mM phosphate buffer of pH 7 and 50 °C after 24, 48, and 72 h represented by the amount of released 2,5-furandicarboxylic acid (FDCA). Each bar represents the average of three independent samples; error bars indicate the standard deviation.

The hydrolytic stability of the sulfonated copolyesters was investigated under neutral conditions at 50 °C in 100 mM potassium phosphate buffer of pH 7 during 72 h. A small increase of released FDCA could be detected over time for five of the six copolyesters (Figure 5). The copolyester POFSI seems to be stable under the tested conditions, which might be due to the low solubility of the polymer. The low solubility of the polymer could be a consequence of the expected microstructure of the ionic polyester in aquatic solvents. The copolyesters are supposed to form nanoparticles in potassium phosphate buffer due to the ionic characteristics of the polyesters. The polyesters with more hydrophobic diols (longer alkyl diols, shorter ether diols) are expected to form bigger particle sizes compared to more hydrophilic diols (shorter alkyl diols, longer ether diols). The polyesters forming bigger particles have less surface area compared to the polyesters forming smaller particles. This could explain the increased hydrolytic stability for polyesters with an increasing length of the diol as well as the higher stability of the ether diol-containing polyesters. The theory is supported by the work of Eisenberg et al. (1990) on random ionomers. According to their paper, the ionic moieties aggregate into “multiplets”, which, in turn, aggregate themselves into “clusters”, finally creating a contiguous phase of restricted mobility in the polymer mass [41]. This would most likely result in a higher hydrolytic stability.

Generally, the glass-transition temperature of the polyester may influence the hydrolytic stability of the polyester. If the hydrolytic degradation is investigated above the glass-transition temperature of the polyester, the polyester chain has an increased mobility and thereby a decreased hydrolytic stability and vice versa at a temperature below the glass-transition temperature of the polyester, as the polyester chain has a decreased mobility and thereby an increased hydrolytic stability (considering only the physical effect of the temperature). However, in this study, the glass-transition temperature of the polyesters does not seem to influence the hydrolytic stability to a large extent. PEFSI (*T*_g_: 84 °C) should be more stable against hydrolytic degradation when compared to POFSI (*T*_g_: 6 °C) from the point of view of the glass-transition temperature, which was, however, not the case. This indicates that the chemical characteristics of the polyesters play a bigger role. Longer alkyl chains and more hydrophobic diols seem to lead to larger particles, resulting in lower solubility and surface area and thereby a higher hydrolytic stability.

Interestingly, in this study, a higher stability was detected for the ether diol-containing copolyesters. Previous work from Bougarech et al. (2014) has demonstrated, contrary to our results, a higher hydrolytic stability for alkyl diol-containing polyesters when compared to ether diol polyesters. This fact was explained with a lower accessibility of the water to the ester bond due to the more hydrophobic nature of the polyester. This could still explain the high hydrolytic stability of POFSI.

Previous studies on the hydrolytic degradation of sulfonated phthalic acid-based copolyesters have indicated that the hydrolysis was due to a nucleophilic substitution of ester groups by water molecules. Chemical architecture as well as accessibility of water molecules towards the copolymer have been mentioned as important parameters, even if it has been demonstrated that the impact of ether diols is subordinate to the impact of the sulfonated moieties [18,36].

Chrisholm et al. (2003) carried out a study of the hydrolytic degradation of sulfonated poly(butylene terephthalate) copolymers, resulting in a comprehensive investigation of the decreased hydrolytic stability of copolyester by introducing 5-sodiosulfoisophthalate units. The authors concluded that the destabilization of the copolyesters is due to a higher water absorption of the polyesters as a consequence of the presence of the ionic group. The increased water absorption is due to the presence of the ionic groups, which results in more hydrophilic polyesters as well as an increased share of amorphous content. Bougarech et al. (2013) demonstrated that the hydrolytic degradation of furanic-sulfonated copolyesters mainly occurs in the regions of the copolymer containing a higher ratio of sulfonated units, assuming block copolyesters, postulating that random copolyesters would display a higher hydrolytic stability.

Carboxylic acid end groups have been shown to have a negative impact on the hydrolytic stability of polyesters due to their catalytic effect, while hydroxyl end groups enhanced the hydrolytic stability [17]. Therefore, the copolyesters investigated in this study were synthesized with an excess of diols to ensure that the hydroxyl end groups enhanced the hydrolytic stability.

### 3.3. Enzymatic Hydrolysis

**Figure 6:**
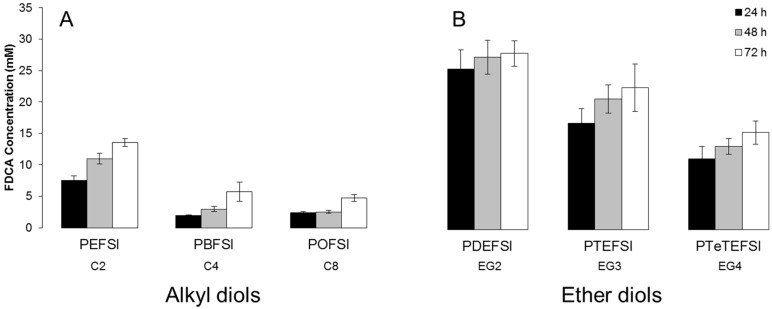
Enzymatic hydrolysis of copolyesters based on 5-sulfoisophthalic acid and 2,5-furandicarboxylic acid with altering (**A**) alkyl diols and (**B**) ether diols by cutinase 1 from *Thermobifida cellulosilytica* after 24, 48, and 72 h at 50 °C represented by the amount of released 2,5-furandicarboxylic acid (FDCA). Each bar represents the average of three independent samples; error bars indicate the standard deviation.

In a next step, the hydrolysis activity of Thc_Cut1 towards the copolyesters based on 5-sulfoisophthalic acid and 2,5-furandicarboxylic acid with altering alkyl and ether diols was investigated. The hydrolysis was performed at 50 °C and pH 7.0, since it has previously been shown to be suitable for PEF hydrolysis [29] as well as for PET hydrolysis with Thc_Cut1 [42]. Thc_Cut1 was proven active towards all of the tested copolyesters, with a decreasing activity with an increasing length of the alkyl diol unit (Figure 6A) as well as for an increasing number of ethylene glycol repeat units (Figure 6B). In all cases, the hydrolytic degradation was low regarding the amount of released FDCA when compared to enzymatic hydrolysis (Figure 5 and Figure 6). The alkyl diol copolyesters showed faster hydrolytic degradation but a lower enzymatic degradation when compared to the ether diol copolyesters. However, an identical trend for hydrolytic and enzymatic degradation cannot necessarily be expected, since the polyesters have to fit in the active site of the enzyme where the polyesters interact with the amino acids in close vicinity. The “presence of oxygen” in the ether diol copolyesters can have an important effect on this enzyme / substrate interaction, and hence determine hydrolysis rates.

Similar results were obtained by Okada et al. (1997) hydrolyzing various furanic polyesters containing aliphatic diols or oligo (ethylene glycols). They observed a decreased hydrolytic activity with lipase from porcine pancreas with an increasing length of the aliphatic diol unit. The results were interpreted as a consequence of the combination of a decreased steric requirement and increased hydrophobicity with increasing length of the diol unit, since a decreased steric requirement facilitates the enzymatic accessibility of the ester linkages while increased hydrophobicity hampers it [43].

In addition, the increased hydrophobicity of polyesters results in decreased water solubility, indicated by the poor water solubility of POFSI, which can also have a negative impact on enzymatic hydrolysis. Increased water solubility has previously also been mentioned as an important parameter to tune and increase the degradation of phthalic esters [44,45]. Eljertsson et al. (1997) concluded that water solubility is a major factor limiting the degradation of hydrophobic phthalic esters by investigating the degradation of phthalic esters under a methanogenic condition. The study clearly shows that phthalic esters with a high water solubility, as for example dibutyl phthalate (DBP), butylbenzyl phthalate (BBP), butyl 2-ethylhexyl phthalate (BEHP), and dihexyl phthalate (DHP), had a higher degradation rate compared to phthalic esters with a lower water solubility, as for example bis(2-ethylhexyl)phthalate (DEHP), dioctyl phthalate (DOP) and didecyl phthalate (DDP). The impact of water solubility on enzymatic hydrolysis has previously been studied for similar polyesters consisting of 1,2-ethanediol and different ratios of terephthalic acid and NaSIP to tune the water solubility and investigate the impact on enzymatic hydrolysis [46,47]. Expectedly, it was shown for different enzymes that the enzymatic hydrolysis decreased with decreasing water solubility [46,47]. This is in line with the data presented in this study, where POFSI with low water solubility was not hydrolyzed by Thc_Cut1.

The increased hydrolysis of water-soluble polymers might be due to the better accessibility of the ester bond for the enzymes. Similarly, an increased flexibility of the chains in polyesters with a low degree of crystallinity seems to facilitate the enzyme to access the ester bonds [37,48]. Several authors have confirmed these findings for the enzymatic hydrolysis of PET with different degrees of crystallinity with various cutinases from *Humilica insolens*, *Pseudomonas mendocina* and *Fusarium solani* [37,49]. Additional reports have demonstrated that amorphous regions are subjected to enzymatic hydrolysis in a higher degree compared to more crystalline regions, which is in line with previous discussions [50,51,52,53]. The low crystallinity of the synthesized copolyesters (below 1%) in this study might therefore have facilitated the enzymatic hydrolysis. An additional indication that chain flexibility plays an important role is the high impact of the temperature difference between a polymer’s melting point and the temperature at which hydrolysis is performed [54]. Considerably higher hydrolysis rates were reported for enzymatic hydrolysis when hydrolysis was performed above the glass-transition temperature of the polymer. In contrast, for the FDCA-based polyesters investigated in this study, the glass-transition temperature seems to have a less significant influence on hydrolysis. Hydrolysis was performed at 50 °C, while those polyesters with the lowest glass-transition temperature of 6 and 14 °C, for the alkyl and ether diol based polyesters, respectively, showed the lowest susceptibility to enzymatic hydrolysis.

In addition, polyesters with ionic moieties are expected to aggregate into “multiplets”, which, in turn, aggregate themselves into “clusters”, finally creating a contiguous phase of restricted mobility in the polymer mass [41]. This microstructure is expected to lead to the low solubility of the polymer. The polyesters with more hydrophobic diols (longer alkyl diols, shorter ether diols) are expected to form bigger particle sizes compared to more hydrophilic diols (shorter alkyl diols, longer ether diols). The polyesters forming bigger particles have less surface area compared to the polyesters forming smaller particles. This could explain the decreased enzymatic hydrolysis towards polyesters with increasing length of the diol and the preferential cleavage of the ester bond of the enzyme.

Another factor influencing enzymatic hydrolysis is the molecular weight of the polyester. Pellis et al. (2016) reported on the enzymatic hydrolysis of PEF with different molecular weights with Thc_Cut1 under the same conditions and reported a released amount of FDCA ranging from 6 to 14 mM after 72 h of incubation with an increasing release of FDCA with increasing molecular weight. This demonstrates that enzymatic hydrolysis can also be affected by the molecular weight of FDCA-containing polyesters. It has generally been believed that the hydrolysis rate decreases with increasing molecular weight [29], but the above results obtained by Pellis et al. (2016) do not support this hypothesis. This previous study compared the enzymatic hydrolysis of PEF in a wide *M*_n_ range from 6 to 40 kDa, while here all FDCA-based polyesters had molecular weights in a more narrow range. Hence, no significant effects are expected on enzymatic hydrolysis. PBFSI, with the lowest *M*_n_, is neither hydrolyzed fastest, confirming the trends reported by Pellis et al. (2016), nor slowest, in agreement with Pellis et al. (2016).

Thc_Cut1 seems to preferably hydrolyze the ester bond in close vicinity to FDCA, as indicated by the absence of NaSIP in the hydrolysis samples. This trend has also been shown for a cutinase and an esterase from *Pseudomonas pseudoalcaligenes* (PpCutA and PpEst) as well as for a putative lipase from *Pseudomonas pelagia* (PpelaLip) hydrolyzing ionic phthalic acid polyesters based on terephthalic acid and NaSIP [46,47]. This is in contrast to the hydrolytic degradation of the copolyesters, where the nucleophilic attack of the water molecules is preferably attacking the ester bond in close connection to the sulfonated group due to the electron drawing effect [18].

**Figure 7:**
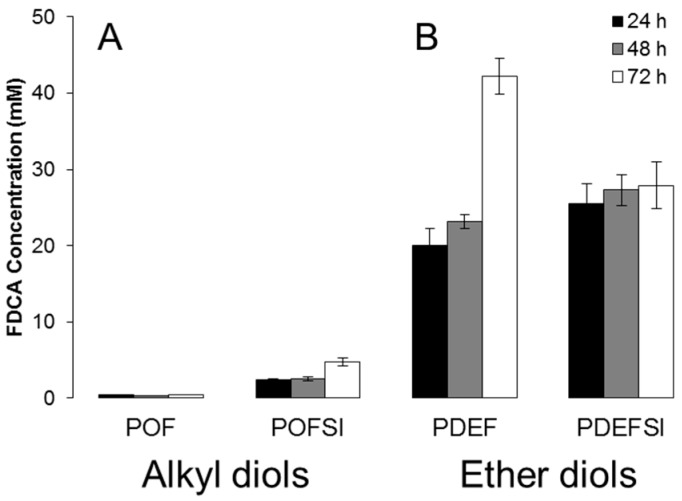
Enzymatic hydrolysis of polyesters based on 2,5-furandicarboxylic acid and copolyesters based on 5-sulfoisophthalic acid and 2,5-furandicarboxylic acid with (**A**) alkyl diol 1,8-octanediol and (**B**) ether diol diethylene glycol by cutinase 1 from *Thermobifida cellulosilytica* after 24, 48 and 72 h at 50 °C represented by the amount of released 2,5-furandicarboxylic acid (FDCA). Each bar represents the average of three independent samples; error bars indicate the standard deviation.

However, Thc_Cut1 does not seem to be negatively affected by the incorporation of sulfonated units in the polyester chain, since the released amount of FDCA corresponded well to the released amount of FDCA for PEF as previously reported [29] as well as for FDCA-based polyesters with altering polyols [30]. Previous studies have also reported on the increased enzymatic hydrolysis of polyesters with an increasing ratio of NaSIP [46,47]. In detail, polyesters based on 1,2-ethanediol and ratios of NaSIP to terephthalic acid of 10:90, 20:80, and 30:70 mol %, were compared and the highest enzymatic hydrolysis was seen for the polyester with the highest ratio of the ionic moiety NaSIP (30:70 mol %) for several enzymes, a cutinase and an esterase from *Pseudomonas pseudoalcaligenes* (PpCutA and PpEst) as well as for a putative lipase from *Pseudomonas pelagia* (PpelaLip) [46,47]. The increased hydrolysis rate was concluded to be a consequence of the increased water solubility. To further evaluate the impact of ionic moieties on enzymatic hydrolysis, polyesters with and without 5-sulfoisophthalic acid were compared (Figure 7). Polyesters based on alkyl diols, represented by POF and POFSI, and polyesters based on ether diols, represented by PDEF and PDEFSI, were evaluated. Indeed, the presence of 5-sulfoisophthalic acid had a positive influence on the enzymatic hydrolysis of the octanediol-based polyester (Figure 7A). As for the ethylene glycol based polyesters, there was a slightly positive influence of 5-sulfoisophthalic acid seen on hydrolysis during 24 h and 48 h, while during prolonged hydrolysis NaSIP seems to have some negative influence (Figure 7B). This is in agreement with the fact discussed above that the enzymes seem to preferentially cleave ester bonds close to FDCA, leading to the accumulation of NaSIP-rich segments, which are then hydrolyzed at lower rates.

**Figure 8:**
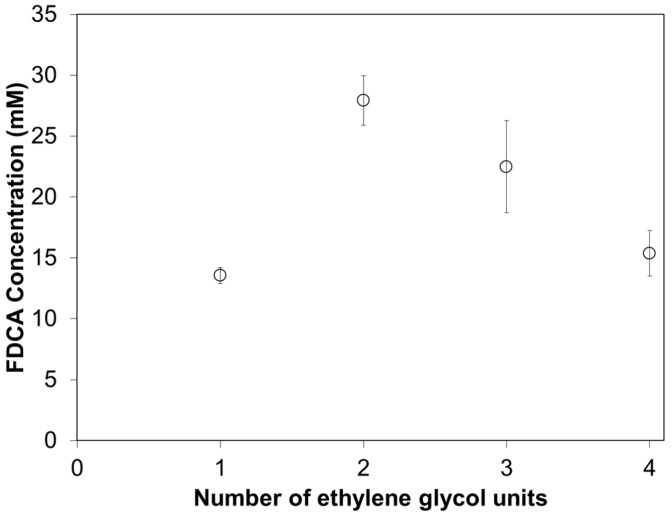
Enzymatic hydrolysis of copolyesters consisting of 5-sulfoisophthalic acid and 2,5-furandicarboxylic acid with altering ether diols by cutinase 1 from *Thermobifida cellulosilytica* after 72 h at 50 °C represented by the amount of released FDCA. Each circle represents the average of three independent samples; error bars indicate the standard deviation.

In addition to increased water solubility as a consequence of the introduction of ionic building blocks in the polyester chain, the introduction of hydrophilic polyols is also of interest. It has previously been suggested by Gigli et al. (2012) to be a suitable parameter to tune polyester hydrolysis [55]. It has also been proven that introducing poly(ethylene glycol) units into polyesters increases hydrolytic degradation as well as enzymatic hydrolysis [30,46,47,56,57,58]. As expected, the enzymatic hydrolysis of the ether diol-based polyesters was significantly increased after 72 h of incubation for the copolyesters PDEFSI and PTEFSI, while the amount of released FDCA was in the same range for PEFSI and PTeTEFSI (Figure 6). In order to evaluate the impact of the ethylene glycol repeat units on enzymatic hydrolysis, copolyesters with one, two, three and four ethylene glycol units were compared (Figure 8). The comparison revealed that the enzymatic hydrolysis for Thc_Cut1 was the highest for polyesters containing two ethylene glycol units (PDEFSI), and continuously decreased when the number of repeats of units was lower or higher. This could indicate that Thc_Cut1 has a preference for shorter polyols and that the balance between the length of the polyol unit and the water solubility has to be tuned to achieve the requested hydrolysis. Haernvall et al. (2017) have previously reported that enzyme hydrolysis increased when replacing the alkyl unit (1,5-pentanediol) by the ether analogue diethylene glycol in FDCA-based polyesters. The replacement doubled the enzymatic hydrolysis rate for Thc_Cut1 and resulted in the release of 103.9 ± 6.3% FDCA after 72 h [30].

**Figure 9:**
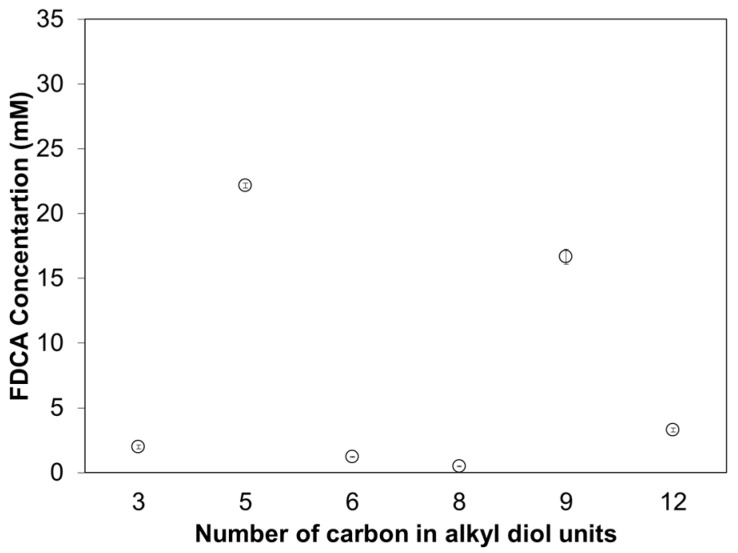
Enzymatic hydrolysis of polyesters consisting of 2,5-furandicarboxylic acid with altering alkyl diols by cutinase 1 from *Thermobifida cellulosilytica* after 72 h at 50 °C represented by the amount of released FDCA. Each circle represents the average of three independent samples; error bars indicate the standard deviation.

The enzymatic hydrolytic mechanism for Thc_Cut1 has further been investigated using FDCA-based polyesters with alkyl diols with altering length (Figure 9). This clearly demonstrates that the polyol length has a great impact on the enzymatic mechanism. Thc_Cut1 shows a preference for polyesters with 1,5-pentanediol and 1,9-nonanediol as alkyl diol moiety. The enzymatic hydrolysis rate does not follow the length of the alkyl diol as it did for the ether diols (Figure 8). Instead, it shows clear peaks for certain chain lengths while the activity is significantly reduced for the length of the units in between, indicating that there are several factors affecting the enzymatic hydrolysis. The chemical composition of the polyesters in turn affects several polymer characteristics, which in turn can affect the enzymatic hydrolysis. For example, there is the odd–even effect [8], where polyesters containing alkyl diols with an odd number of methylene groups have lower melting temperatures compared to polyesters containing alkyl diols with an even number of methylene groups. This effect can explain the trend seen for Thc_Cut1 hydrolyzing the alkyl diol-based polyesters, where the highest activities were seen for the alkyl units with an odd number of carbon (Figure 8). The enzymatic hydrolytic mechanism for Thc_Cut1 has also previously been investigated [30]. Haernvall et al. (2017) investigated how the polyol structure influences Thc_Cut1 hydrolysis of bio-based 2,5-furandicarboxylic acid (FDCA) polyesters. Their study confirmed by ^1^H-NMR analysis that Thc_Cut1 did not release any oligomeric hydrolysis products but that Thc_Cut1 only released monomeric release products hydrolyzing FDCA-based polyesters. The influence of the chemical composition of the polyesters on enzymatic hydrolysis has recently also been investigated for different enzymes with similar polyesters containing NASIP and terephthalic acid instead of FDCA [30,46,47].

## 4. Conclusions

A series of partially bio-based copolyesters consisting of 5-sulfoisophthalic acid and 2,5-furandicarboxylic acid with altering alkyl and ether diols were successfully synthesized and characterized. In addition, the hydrolytic degradation and enzymatic hydrolysis of the copolyesters under ambient conditions and by cutinase 1 from *Thermobifida cellulosilytica* (Thc_Cut1) was investigated. The hydrolytic stability was increased with an increase in the length of the alkyl diol unit, while it was reduced by increasing the length of the ether diol unit. The hydrolytic stability of the sulfonated polyester is probably due to the increased access of water molecules with increasing hydrophilicity. Thc_Cut1 was able to hydrolyze FDCA-based polyester based on alkyl diols ranging from two to eight hydrocarbon chain lengths, while the highest activity was detected for the shorter chain lengths. Increased hydrolysis was observed by exchanging an alkyl diol to ether diols. A positive influence of introducing an ionic phthalic acid in the backbone has been seen on enzymatic hydrolysis even though the enzyme clearly preferred the cleavage of ester bonds in vicinity to the non-charged building blocks. The improved knowledge about the synthesis, hydrolytic degradation, and enzymatic hydrolysis of bio-based furanic-sulfonated isophthalic copolyesters is important for the expected development within polymer science towards an increasing share of bio-based polymers and environmentally friendlier processes. This improved knowledge can be used for developing environmentally friendlier functionalization alternatives for FDCA-based polyesters as well as biotechnological recycling strategies.

## Figures and Tables

**Table 1:** Properties of the copolyesters based on 5-sulfoisophthalic acid and 2,5-furandicarboxylic acid with various alkyl and ether diols measured by GPC analyses, and the thermal properties of the FDCA-based polyesters determined by DSC analysis.

Group	Polyol	Code	Composition ^a^		GPC ^b^		DSC ^e^
			Feed	Copolyester			
			(F):(SI) (mol %)	(F):(SI) (mol %)	*M*_n_^c^ × 1000 (g mol^−1^)	PDI ^d^	*T*_g_^f^ (°C)
Alkyl diol	1,2-Ethanediol	PEFSI	70:30	-	3.28	1.4	84
	1,4-Butanediol	PBFSI	70:30	60:40	2.63	1.3	57
	1,8-Octanediol	POFSI	70:30	62:38	5.79	2.3	6
Ether diol	Diethylene glycol	PDEFSI	70:30	69:31	6.83	1.9	61
	Triethylene glycol	PTEFSI	70:30	70:30	8.03	2.1	29
	Tetraethylene glycol	PTeEFSI	70:30	69:31	6.96	2.0	14

^a^ Furanoate (F) to 5-sodiosulfoisophtalate (SI) molar ratio in the initial reaction mixture and in the copolymer determined by ^1^H-NMR. ^b^ Gel permeation chromatography performed on crude samples using dimethylacetamide as a solvent. ^c^ *M*_n_: number average molecular weight. ^d^ PDI: polydispersity index. ^e^ DSC was performed from 80 to 200 °C with one heating and cooling run at 20 °C/min. ^f^ *T*_g_: Glass-transition temperature.

**Table 2:** FTIR data of the copolyesters based on 5-sulfoisophthalic acid and 2,5-furandicarboxylic acid with altering polyols, where the codes represent the different alkyl or ether diols in the copolyesters.

Polyol	Code	Assignment (cm^−1^)									
		=CH (Furan)	C–H (CH2)	C=O (ester)	C=C (Furan)	C–O (ester)	Furan Ring Breathing	2,5-Disubstituted Furan Ring	SO2 Asymmetric Stretching Vibrations	SO2 Symmetric Stretching Vibrations	S–O
1,2-Ethanediol	PEFSI	3127	2960	1716	1582	1269	1020	966,827,763	1047	1127	753
1,4-Butanediol	PBFSI	3128	2958	1713	1582	1274	1021	967,827,765	1049	1134	754
1,8-Octanediol	POFSI	3121	2976	1717	1578	1272	1017	967,820,766	1048	1139	754
Diethylene glycol	PDEFSI	3121	2957	1717	1581	1272	1019	965,827,763	1047	1117	753
Triethylene glycol	PTEFSI	3122	2875	1717	1582	1272	1021	964,827,764	1047	1104	753
Tetraethylene glycol	PTeEFSI	3121	2874	1717	1582	1273	1022	963,831,765	1047	1102	755
